# A Bionic Polarization Navigation Sensor and Its Calibration Method

**DOI:** 10.3390/s16081223

**Published:** 2016-08-03

**Authors:** Huijie Zhao, Wujian Xu

**Affiliations:** School of Instrument Science and Opto-Electronics Engineering, Beihang University, No. 37 Xueyuan RD, Haidian, Beijing 100191, China; hjzhao@buaa.edu.cn

**Keywords:** polarization, navigation, mathematical model, calibration

## Abstract

The polarization patterns of skylight which arise due to the scattering of sunlight in the atmosphere can be used by many insects for deriving compass information. Inspired by insects’ polarized light compass, scientists have developed a new kind of navigation method. One of the key techniques in this method is the polarimetric sensor which is used to acquire direction information from skylight. In this paper, a polarization navigation sensor is proposed which imitates the working principles of the polarization vision systems of insects. We introduce the optical design and mathematical model of the sensor. In addition, a calibration method based on variable substitution and non-linear curve fitting is proposed. The results obtained from the outdoor experiments provide support for the feasibility and precision of the sensor. The sensor’s signal processing can be well described using our mathematical model. A relatively high degree of accuracy in polarization measurement can be obtained without any error compensation.

## 1. Introduction

Polarization is one of the basic attributes of light which contains a lot of specific information. Partially-polarized light is ubiquitous in nature. It is imperceptible to humans but can be detected by quite a number of insects. Scientists have discovered that the direction information of this polarized skylight can be exploited by these insects for navigation or course control. For example, the desert ant *Cataglyphis*, the honeybee and the field cricket show special abilities in polarization navigation skills during their foraging and homing [1,2,3,4,5]. The dung beetle can even utilize the dim and partially polarized lunar skylight at night for orientation [6,7]. The number of polarization navigation cases in nature is so numerous, that we cannot enumerate all of them here. The polarized skylight can also be used by humans for navigation. It has been hypothesized that the Vikings (between AD 900 and AD 1200) might have been able to determine the solar azimuth angle by watching the polarized skylight, just like some insects [8,9]. The Vikings could have used the skylight compass with the help of a ‘sunstone’, which functioned as a linearly polarizing filter. With the development of technology, the working principles of polarization navigation have been better understood and employed by people. Scientists have researched in this field for decades and significant achievements have been reached in the study of skylight polarization patterns and bionic navigation sensors.

There are two basic requirements in insects’ polarization navigation. The first is the relatively stable skylight polarization pattern, which is mainly decided by the solar position and atmospheric conditions. The second is the structure and function of the compound eye and specialized visual nervous system which supports the recognition of the direction from partially polarized skylight.

**Figure 1:**
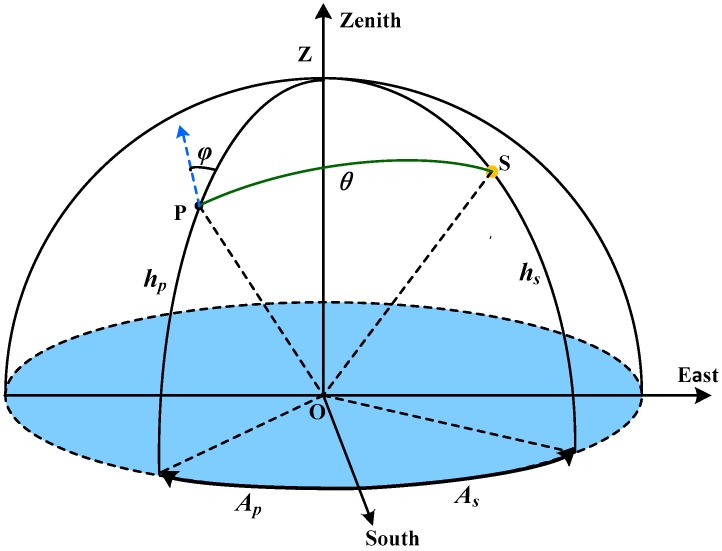
The angle of polarization (AOP) of an arbitrary scattered skylight as experienced by an observer in point O. S stands for the solar position and Z stands for the zenith. P stands for the direction of scattered light. $h_{s}$ and $h_{p}$ stands for the elevation angle of sun and skylight, respectively. $A_{s}$ and $A_{p}$ stands for the azimuth angle of sun and skylight, respectively. θ stands for the scattering angle. φ stands for AOP, which is the angle between the polarization direction (the blue arrow) and the reference plane (OPZ).

Direct sunlight is unpolarized but becomes partially polarized after scattering by the particles in the atmosphere. Since most particles in the atmosphere are gas molecules, the sizes of which are much smaller than the wavelength of visible sunlight, the scattering process can be described as the single-scattering Rayleigh model [10]. The Rayleigh scattering and the local density fluctuations of the atmosphere lead to the polarization of scattered light [11]. Rayleigh scattering light has two significant properties which decide the polarization patterns of skylight. Firstly, the polarizing direction of scattered light is perpendicular to the scattering plane. Secondly, the degree of polarization (DOP) is related to the scattering angle, i.e., the DOP will increase when the scattering angle increases from 0° to 90°, and it will decrease when the scattering angle increases from 90° to 180°. The highest DOP of skylight is usually concerned with the solar zenith angle and the conditions of atmosphere. After analyzing a series of measured DOP data sets of clear skylight, Coulson summarized a semi-empirical Rayleigh scattering model which gives the estimation of the highest DOP at different solar zenith angles [12]. The angle of polarization (AOP) for an arbitrary scattered beam of light was defined in Figure 1. The observing point on the ground was set to be the original point “**O**”. The horizontal coordinate system was chosen.

The scattering angle (θ) is the angle between the solar beam (SO) and the scattering beam (PO). The relationship of the scattering angle and other components can be expressed as Equation (1).

(1) $$\cos\theta =\sin(h_{p})\sin(h_{s})+\cos(h_{p})\cos(h_{s})\cos(A_{s}-A_{p})$$

The relationship between the AOP (φ) and other components can be expressed as Equation (2).

(2) $$\cos\varphi =\frac{\sin(\left|A_{s}-A_{p}\right|)}{\sin\theta }\cos(h_{s})$$

A 3-D representation of the pattern of polarization can be found in Wehner’s work [13]. The polarization patterns of skylight are relatively stable and predictable under clear sky conditions. The solar azimuth angle can be derived from the polarized skylight and the direction information can be derived further. This is the basic principle utilized by these insects and Vikings for polarization navigation.

The semi-empirical Rayleigh scattering model agrees well with the polarization patterns observed in a clear sky [14]. However, in common conditions, the clouds and aerosols are usually inevitable, which bring large particles in the scattering process. When light is scattered by large particles, the Mie scattering and multiple scattering usually dominate. The polarization patterns of skylight will be modified and become different from the semi-empirical Rayleigh model. The Mie scattering and multiple scattering could depolarize the skylight and significantly reduce the DOP of the scattering light. The magnitude of the pattern would be modified by atmospheric effects; however, the direction of polarization can be stable under most conditions [15,16]. The stability of the dominant AOP of skylight makes it possible to use a skylight compass not only in clear sky conditions but also in cloudy or hazy weather. Henze and Labhart demonstrated that the field crickets can rely on skylight polarization even under unfavorable celestial conditions, emphasizing the significance of polarized skylight orientation for insects [4]. Of course, the polarization patterns of skylight could be totally destroyed when the weather is extremely bad, such as rain, sand storm, etc. This paper is mainly concerned with the polarization navigation under the clear sky conditions and focuses on the design of the sensor.

The insects’ ability of recognizing the direction from partially polarized skylight is mostly achieved via specialized regions of the compound eye and polarization-opponent neurons (POL-neuron). For example, the retinulae in the dorsal rim area (DRA) of a field cricket are oriented in different directions and sensitive to different AOPs. The optic lobe POL-neurons of field crickets could analyze and recognize the AOP [17,18]. Inspired by the polarization navigation principles of insects, scientists have developed kinds of polarization navigation sensors which could be used in the mobile robots and unmanned aerial vehicles. Labhart and his co-workers developed two mobile robots, Sahabot and Sahabot2, and achieved success in the navigation experiments using the polarization navigation sensor with six channels [19,20]. The differently oriented polarizers in each channel imitate the function of the differently oriented retinulae of the insect’s compound eye. The electronic components modeled the function of the optic lobe POL-neurons of field crickets. The orientation of the mobile robot could be calculated with a microcontroller which analyzes the information of polarization, time and position. Some other polarization sensors were excogitated after Labhart with several improvements that make it more suitable for navigation. Chu and colleagues [21] and Fan and colleagues [22] proposed two polarization sensors with six and four channels, respectively, which improved the levels of accuracy and integration. Some calibration works were implemented in their systems to reduce the measurement errors. However, the error sources were not fully considered and the offset of the error components were not figured out. Chahl and Mizutani developed and flight-tested a sky polarization compass whose accuracy was found to be comparable to a solid state magnetic compass [23]. The polarization compass was proved to be meaningful in the navigation of the unmanned aerial vehicles. Some camera based skylight compasses were developed by Usher, Horváth, Carey, Zhang and others that used cameras and linear polarizing filters to acquire polarization photos of the sky dome [24,25,26,27]. The navigation information could be derived from these polarization photos after image processing. Most of the camera based polarization sensors were designed to detect the polarization patterns of sky. Since it is not easy to derive the orientation information from photos, this kind of sensor was seldom applied to mobile robots or vehicles. Sarkar and others developed an integrated polarization analyzing a CMOS image sensor for navigation. The computation of the Stokes parameters could be implemented on-chip. This kind of design would greatly miniaturize the navigation sensor [28]. Gruev and his co-workers developed a CCD polarization imaging sensor [29]. However, the fabrication processes of the integrated CMOS/CCD sensors call for special manufacturing technology and the accuracy would be affected by the instantaneous-field-of-view (IFOV) errors, the nonuniformity between different detectors and unresponsive or dead pixels [30]. Yet the IFOV errors would not be problems when detecting the sky dome since the scattered skylight is usually homogeneous in a small area [12]. Chu and his co-workers developed an integrated polarization dependent photodetector, which improved the detection accuracy and made the sensor much smaller [31]. The sensor designed by Chu and colleagues had some similarities with the CCD/CMOS designs, but it was a non-imaging sensor. Since the error sources of the sensor were not calibrated, the accuracy of the sensor was improved by error compensation. However, the compensation results might be affected by the variance of the incident light. Lu and others developed a single channel polarization imaging system with a fast rotating polarizer. Though the number of channels was reduced, the mechanical structure and control system became more complicated and the real-time performance was not as good as in the multi-channel structure [32].

The bionic sensor proposed in this paper is a basic polarization detecting unit which is modeled the function of a single retinula and POL-neuron of the insect. It adopts the multi-channel structure which is a non-imaging design. Differently orientated polarizers act as the AOP analyzer and the photodiodes act as the light intensity detector. This is a classical structure of polarization navigation sensor which draws on the experiences of the previous designs. This kind of ‘point-source’ design is suitable for polarization navigation, and its accuracy has been proved to be higher than other designs. We have tried to make the size of our sensor smaller than the previous designs. In addition, the design is easy to expand to a multi-sensor system for future applications. The optic designs have been proposed by us in this paper together with the signal processing and polarization resolving methods. In addition, this paper pays much attention to the calibration method. Different interference factors have been taken into consideration, such as the mounting error of the polarizer, the response error of the photodiode and the amplification error of the electric circuit. A more detailed mathematical model has been built which agrees well with the experimental data. The variable substitution and nonlinear curve fitting method were used in the calibration process and have been proved to be of high efficiency and accuracy.

## 2. Materials and Methods

### 2.1. The Design of Polarization Navigation Sensor

**Figure 2:**
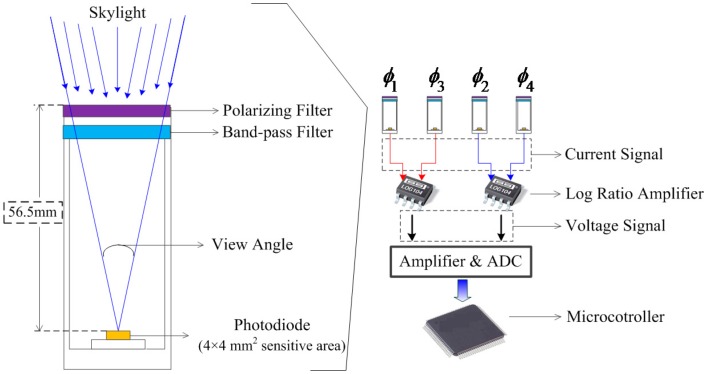
Left part: The sketch map of a single channel; Right part: The integral structure of the sensor.

The sensor proposed in this paper is a parallel four-channel detector. Each channel contains a linear polarizer, a band-pass filter and a photodiode. The sketch map of a single channel is shown in Figure 2 (left part). The polarizers are oriented in four directions, i.e., $\left(\phi_{1},\phi_{2},\phi_{3},\phi_{4}\right)$, relative to the 0° reference direction. The channel $\phi_{1}$ is orthogonal to $\phi_{3}$, channel $\phi_{2}$ is orthogonal to $\phi_{4}$. The orthogonal structure modeled the polarization-opponent neurons of insects which receive antagonistic input from two polarization-sensitive channels with orthogonal AOPs [19]. The polarizers are mounted in front of the sensor, so that the AOPs of incident light will not be affected by the filters or other complex lens. Consequently, the polarization states of incident light can be correctly detected. Some other designs usually set the band pass filter in front of the linear polarizer. For the integrated polarization analyzing CMOS/CCD image sensor, the lens and filters in front of the sensors might change the AOPs of incident light.

The extinction ratio of the linear polarizer is 500:1 with relatively high transmittance. Figure 2 (right part) shows the basic working process of our sensor. Firstly, current signals increase from the four photodiodes when receiving scattering skylight. Secondly, the two groups of current signals are turned into two voltage signals by the log ratio amplifiers. Here we chose the LOG104 amplifiers manufactured by Texas Instruments. After voltage amplification the analog signals are turned into digital signals before further calculations in the microcontroller. The microcontroller we chose here is a field programmable gate array (FPGA) module produced by Altera.

In the polarization navigation, the partially polarized skylight is the reference target which needs to be detected and exploited by our sensor. The polarization states vary as a function of different incident directions. In order to identify the polarization states of a certain direction, the field of view (FOV) of the sensor should be as small as possible so that the detected sky area is limited to a “point”. However, an insufficiently large FOV would lead to a decrease of the signal noise ratio (SNR), because the energy of incident light would be too weak. (The actual SNR of our sensor ranges from 50 to 100, which is affected by the intensity of incident light and the degree of polarization.) The longer integration time will cause a loss to the real-time performance. After a balance between the incident energy and detecting area, the FOV of our sensor was set to 10°. On one hand, it can satisfy the sensor’s requirement of the intensity of incident light and the SNR can meet a high standard. On the other hand, the 10° FOV corresponds to an area of 0.024 sr solid angle in the sky hemisphere, among which the skylight is evenly distributed in both intensity and polarization. The skylight radiance distribution model proposed by Harrison and Coombes [33] can be used to model the intensity of skylight. The intensity usually changes significantly around the solar position. However, it changes slowly at other points. According to the Rayleigh scattering theory, the DOP of scattered skylight around the sun is relatively low, so that is not appropriate for polarization navigation. So the polarization navigation sensor usually detects the sky area with high DOP where the intensity is evenly distributed. As for the AOP of skylight, it is known that the AOP varies from point to point even in a small sky area. The polarization direction at the edge of FOV could be several degrees different from that of the central point. This variance cannot be neglected. However, the polarization directions in the FOV are symmetric about the solar meridian. The combination of the skylight in the FOV will result in the polarization direction being aligned with that of the central point. The detection result is the average of the FOV area, so it is a kind of “point-source” sensor.

**Figure 3:**
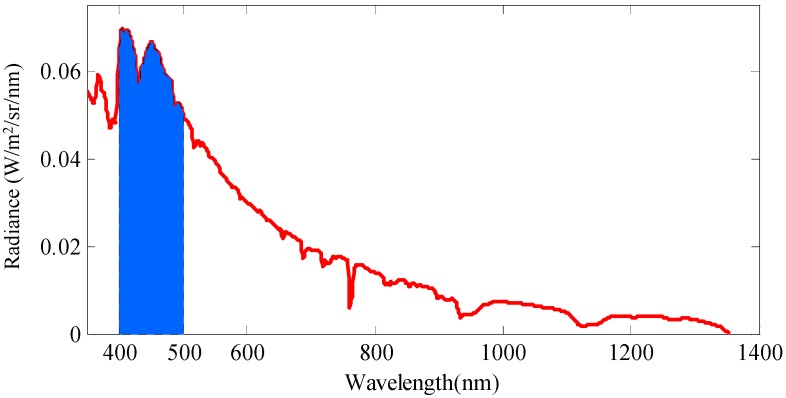
The spectral radiance curve of the clear skylight measured with an analytical spectral device (ASD). The field-of-view (FOV) of the ASD is 25°. The measurement was carried out at 8:10 (UTC) on 30 May 2013 in Beijing, China. The zenith area of skylight was detected while the solar zenith angle was about 53°.

In Rayleigh scattering, the intensity of scattered light is inversely proportional to the biquadrate of the wavelength. So the blue light (short wavelength) usually dominates the skylight in the daytime, and that is why the sky looks blue. To further investigate the scattered light of clear sky, we took a spectral measurement of a certain clear sky area (without clouds or direct sunlight) using an analytical spectral device (ASD) manufactured by PANalytical Company (Boulder, CO, USA). The spectral radiance curve is shown in Figure 3. Although the spectral data may be variable in a different time, position and direction, the spectral features in Figure 3 are authentic. The intensity of skylight varies with incident direction. Generally speaking, the closer to the solar position, the brighter the skylight is.

From Figure 3 we can find that the violet blue band has relatively high intensity which corresponds with the Rayleigh theory. So our sensor takes a 400–500 nm band-pass filter to choose the incident light band which is painted blue in Figure 3. Beside intensity, the selection of spectral range corresponds more or less to the spectral sensitivity of the polarization sensitive blue receptors in the POL area of crickets [5]. The violet blue sensitive photodiodes are chosen correspondingly, of which spectral responses are about 0.25 A/W in the chosen band. The chosen photodiodes are QY-S114QM manufactured by Qingyue Tech (Shanghai, China). The photodiodes increase current signal when receiving irradiation. The ideal response of a photodiode can be expressed as Equation (3).

(3) $$S_{i}=\frac{1}{2}KI\left[1+d\cos\left(2\phi -2\phi_{i}\right)\right]$$

where the parameter $S_{i}$ is the output current of photodiode i, K is the response factor which describes the spectral sensitivity (unit: A/W), I is the intensity of light arriving at the active area of the photodiode, d is the DOP of incidence, ϕ is the angle of polarization (AOP) of incident light relative to the sensor’s 0° direction, $\phi_{i}$ is the orientation of polarizer in channel i.

$\phi_{1},\phi_{2},\phi_{3},\phi_{4}$ are designated to 0°,45°,90°,135°, respectively. Channel 1 and 3 form a pair of orthogonal paths, channel 2 and 4 form another pair of orthogonal paths. The two sets of response currents produced by an orthogonal group will be turned into voltage signal after being processed by a log ratio amplifier. The log ratio amplifier corresponds to the action of insect’s POL-neuron which enhances AOP contrast sensitivity and makes the AOP response insensitive to fluctuations of light intensity. The working process is intelligibly shown in Figure 2 (right part). The output voltage of the two pairs of orthogonal paths can be briefly expressed as Equation (4).

(4) $$\left\{ \begin{array}{l} p_{1}=\frac{1}{2}\log\frac{S_{1}}{S_{3}}\\ p_{2}=\frac{1}{2}\log\frac{S_{2}}{S_{4}} \end{array}\right.$$

Through Equations (3) and (4) we can derive the calculation formulas of DOP and AOP of incident light, as shown in Equation (5). *t*_1_ and *t*_2_ are two intermediate variables.

(5) $$\left\{ \begin{array}{l} t_{1}=10^{2p_{1}}\\ t_{2}=10^{2p_{2}}\\ \tan(2\phi )=\frac{\left(t_{2}-1)\cos(2\phi_{1})+(1-t_{1})\cos(2\phi_{2})+(t_{1}-t_{1}t_{2})\cos(2\phi_{3})+(t_{1}t_{2}-t_{2})\cos(2\phi_{4}\right)}{\left(1-t_{2})\sin(2\phi_{1})+(t_{1}-1)\sin(2\phi_{2})+(t_{1}t_{2}-t_{1})\sin(2\phi_{3})+(t_{2}-t_{1}t_{2})\sin(2\phi_{4}\right)}\\ d=\frac{t_{1}-1}{\cos(2\phi -2\phi_{1})-t_{1}\cos(2\phi -2\phi_{3})} \end{array}\right.$$

During the calculation of DOP and AOP, the error items have been ignored in the above equations. However, in a real situation the error is inevitable, e.g., the polarizer mounting errors, photodiode response errors and electric circuit noises. The true mathematical model of the sensor is much more complicated. The detection results will be unreliable without calibration or error compensation. In the next section, an improved model which considers these error terms is introduced, and the calibration method is proposed.

### 2.2. Calibration Method

It is important to calibrate this kind of sensor before it can be used, because there are many error sources from both the optical devices and circuits. Many other systems that perform similar functions did not carry out this calibration step [19,20,34]. The most commonly used calibration method was the least square based calculation algorithm. Zhao [35] presented an error compensation algorithm, based on a least square support vector machine. However, it only performed output angle error compensation according to the input AOP, which did not take the real error sources into consideration. Xian [36] presented a least squares based calculation algorithm by employing all outputs of the sensor. Much more error sources had been considered and a better calibration result had been achieved compared with these former methods. The calibration method presented in this paper considers most of the error sources, and the complete mathematical model of the sensor was built and simplified, which made the calibration simpler. In addition, Xian’s method utilized the information from six channels, but only four channels were used in our method. The error sources and curve fitting method are introduced in this section.

During the construction of the polarization navigation sensor, the linear polarizers in each channel could not be mounted in the exact orientation as designed. The value of $\phi_{1},\phi_{2},\phi_{3},\phi_{4}$ might usually be offset by about a few degrees, which will cause great errors in the calculation of AOP and DOP. So the true value of $\phi_{i}$ needs to be ascertained. In addition, the errors from photodiode, log ratio amplifier and signal processing circuit cannot be neglected. The response current of the four photodiodes in practice is as Equation (6) (compare with Equation (3)).

(6) $$\left\{ \begin{array}{l} S_{1}=\frac{1}{2}K_{1}I\left[1+d\cos\left(2\phi -2\phi_{1}\right)\right]+D_{1}\\ S_{2}=\frac{1}{2}K_{2}I\left[1+d\cos\left(2\phi -2\phi_{2}\right)\right]+D_{2}\\ S_{3}=\frac{1}{2}K_{3}I\left[1+d\cos\left(2\phi -2\phi_{3}\right)\right]+D_{3}\\ S_{4}=\frac{1}{2}K_{4}I\left[1+d\cos\left(2\phi -2\phi_{4}\right)\right]+D_{4} \end{array}\right.$$

Because of the difference between the four photodiodes, the response factors $K_{1},K_{2},K_{3},K_{4}$ are different from each other. The extinction coefficient of each channel is also treated as a factor multiplied in $K_{i}$. $D_{1},D_{2},D_{3},D_{4}$ are the dark currents of the four photodiodes. The log ratio amplifiers have output offset voltage errors and gain accuracy errors. The signal processing circuit also has gain accuracy errors. The actual output voltage of the two sets of orthogonal paths is shown in Equation (7) (compare with Equation (4)).

(7) $$\left\{ \begin{array}{l} p_{1}=R_{1}\left\{\frac{1}{2}\Delta_{1}\log\left(\frac{\frac{1}{2}K_{1}I\left[1+d\cos\left(2\phi -2\phi_{1}\right)\right]+D_{1}}{\frac{1}{2}K_{3}I\left[1+d\cos\left(2\phi -2\phi_{3}\right)\right]+D_{3}}\right)+V_{OSO1}\right\}\\ p_{2}=R_{2}\left\{\frac{1}{2}\Delta_{2}\log\left(\frac{\frac{1}{2}K_{2}I\left[1+d\cos\left(2\phi -2\phi_{2}\right)\right]+D_{2}}{\frac{1}{2}K_{4}I\left[1+d\cos\left(2\phi -2\phi_{4}\right)\right]+D_{4}}\right)+V_{OSO2}\right\} \end{array}\right.$$

$\Delta_{1},\Delta_{2}$ are the gain factors of the two log ratio amplifiers. The theoretical value of $\Delta_{1},\Delta_{2}$ are both 1. $V_{OSO1},V_{OSO2}$ are the output offset voltages of the two log ratio amplifier of which typical value are 3 mV. $R_{1},R_{2}$ are the amplification factors of the signal processing circuit which are designed as 2. When measuring the skylight, the current signals raised by the photodiodes are higher than 1 μA after considering the extinction of the polarizer and the band-pass filter. However, the dark current of the photodiode is lower than 0.5 nA which is far less than the response current. So the dark currents of photodiodes are ignored in the calibrating calculation.

In summary, the factors need to be calibrated including $R,\Delta ,V_{OSO},K,\phi_{i}$, and there are all together 14 unknown parameters. From the mathematical model (Equation (7)) of the sensor we can find that it is difficult to measure the unknown parameters directly. Besides, there are many correlations between different parameters which make it more difficult to acquire the accurate values of each parameter. This paper has made an overall consideration over the sensor model and the different error features, the method of parameter substitution and curve fitting was applied in the calibration of the sensor. The method is carried out in the following steps.

Firstly, expressions of the output voltages in Equation (7) can be transformed into Equation (8).

(8) $$\left\{ \begin{array}{l} p_{1}=\frac{1}{2}\Delta_{1}R_{1}\log\frac{K_{1}}{K_{3}}+R_{1}V_{OSO1}+\frac{1}{2}\Delta_{1}R_{1}\log\frac{1+d\cos\left(2\phi -2\phi_{1}\right)}{1+d\cos\left(2\phi -2\phi_{3}\right)}\\ p_{2}=\frac{1}{2}\Delta_{2}R_{2}\log\frac{K_{2}}{K_{4}}+R_{2}V_{OSO2}+\frac{1}{2}\Delta_{2}R_{2}\log\frac{1+d\cos\left(2\phi -2\phi_{2}\right)}{1+d\cos\left(2\phi -2\phi_{4}\right)} \end{array}\right.$$

It is obvious that the output voltages periodically change with the variation of ϕ (the AOP of incident light). Then the parameter substitution is adopted to simplify the formula as Equation (9).

(9) $$\left\{ \begin{array}{l} B_{1}=\frac{1}{2}\Delta_{1}R_{1}\log\frac{K_{1}}{K_{3}}+R_{1}V_{OSO1}\\ A_{1}=\frac{1}{2}\Delta_{1}R_{1}\\ B_{2}=\frac{1}{2}\Delta_{2}R_{2}\log\frac{K_{2}}{K_{4}}+R_{2}V_{OSO2}\\ A_{2}=\frac{1}{2}\Delta_{2}R_{2} \end{array}\right.$$

The new expression of the output voltage is shown in Equation (10).

(10) $$\left\{ \begin{array}{l} p_{1}=B_{1}+A_{1}\log\frac{1+d\cos\left(2\phi -2\phi_{1}\right)}{1+d\cos\left(2\phi -2\phi_{3}\right)}\\ p_{2}=B_{2}+A_{2}\log\frac{1+d\cos\left(2\phi -2\phi_{2}\right)}{1+d\cos\left(2\phi -2\phi_{4}\right)} \end{array}\right.$$

where $B_{1},B_{2}$ could be considered as the offset voltages which are both designated to a value of 0. $A_{1},A_{2}$ could be treated as the amplification factors with theoretical values of 1 (decided by the gain factors of the two log ratio amplifiers and the amplification factors of the signal processing circuit). Equation (10) is greatly simplified compared with Equation (8). The parameters to be calibrated become $A_{1},A_{2},B_{1},B_{2}$ and four polarizer orientation angles $\phi_{1},\phi_{2},\phi_{3},\phi_{4}$. There are now all together eight parameters that need to be identified, and the other six parameters have been reduced from the original model.

The calculation of AOP (ϕ) and DOP (d) of incident light is the same as Equation (5) except for the calculation of the two intermediate variables $t_{1}$ and $t_{2}$, which are shown in Equation (11).

(11) $$\left\{ \begin{array}{l} t_{1}=10^{(p_{1}-B_{1})/A_{1}}\\ t_{2}=10^{(p_{2}-B_{2})/A_{2}} \end{array}\right.$$

In order to figure out the sensor’s unknown parameters, we used linearly polarized light as the reference light source of which AOP and DOP were known and could be accurately controlled. A series of output voltage signal $p_{1},p_{2}$ were collected at different incident AOPs. Then the curve fitting was applied according to the measured data sets and Equation (10), so that an optimized estimation on the above parameters can be obtained.

### 2.3. Calibration Experiment

**Figure 4:**
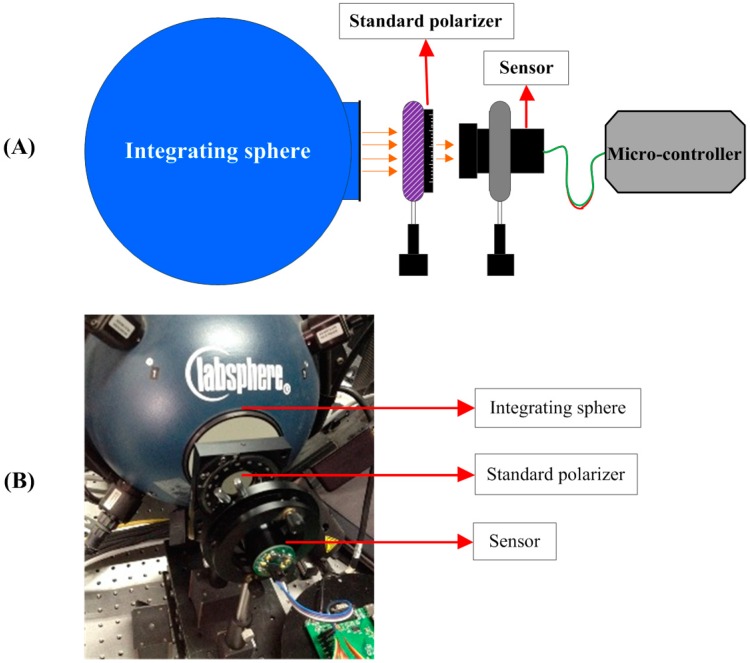
(**A**) The sketch map of the calibration experiment; (**B**) The photo of the calibration experiment. The integrating sphere was manufactured by Labsphere, and the light sources coupled to the integrating sphere are three halogen lamps for general purpose. The linear polarizer was a product of Meadowlark Optics. The linear polarizer can be manually rotated to any direction with the help of a precise gear driving device mounted on the holder of the polarizer. The rotating accuracy is ±2 arc minute.

An integrating sphere manufactured by Labsphere (North Sutton, NH, USA) was used as the light source in the calibration experiment. The luminance of the integrating sphere was adjusted to the similar level of the clear skylight. The light from an integrating sphere was unpolarized, so we set a linear polarizer in front of the out port of the integrating sphere. The linear polarizer used here was manufactured by Meadowlark Optics (Frederick, CO, USA) of which the polarization orientation can be accurately rotated and the rotating accuracy was ±2 arc minute. The linear polarizer can be manually rotated to any direction with the help of a precise gear driving device mounted on the holder of the polarizer. The holder and the gear driving device used to rotate the polarizer were manufactured by Meadowlark Optics, too. The light from the integrating sphere became linearly polarized after going through the linear polarizer. The DOP and AOP of the outgoing light were decided by the polarizer. After receiving the incident light, response current signals increased in the photodiodes, the further process and calculation was carried out in the signal processing circuit and the microcontroller. Figure 4A shows the diagrammatic sketch of the experiment and Figure 4B shows the scene of the calibration experiment.

**Figure 5:**
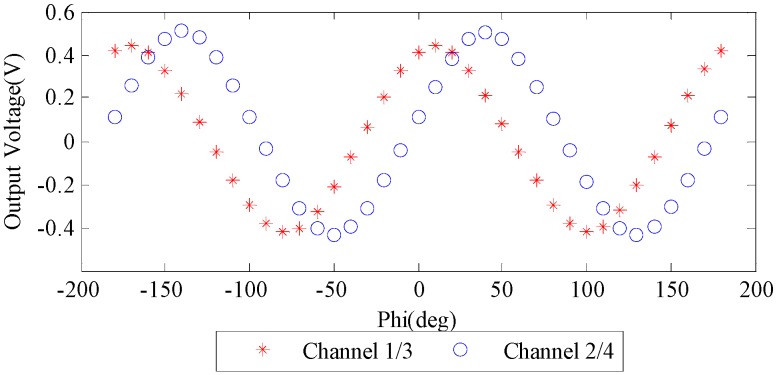
The output voltage of the two sets of orthogonal paths. The horizontal axis ‘Phi’ represents the AOP (ϕ) of incident light.

In the calibration experiment, we firstly set the 0° of the standard polarizer to align with the sensor’s reference direction. At this time, the AOP of incident light was 0° (ϕ=0). The sensor automatically collected a set of output voltage data at a certain incident AOP. Then we rotated the linear polarizer with an angle step of 10°. The voltage data was collected at each rotating step. After a full circle rotation, a series data of incident AOP and output voltage was obtained. Figure 5 shows the relationship between the voltage and AOP. The variation period of this curve is 180°, because the polarizer returned to the prime direction after it rotated by 180°. So it is sufficient to concern ourselves with the data collected in one period, i.e., from −90° to 90°.

The output voltage in Figure 5 is close to what would be predicted by Malus’ law. The relationship between them could be expressed as Equations (3) and (4). Overall, the output voltage agrees well with the theoretic model, but some details illustrate the influence of the error sources, for example, the phase shifting of the two groups of curves, and the differences of amplitudes. Moreover, many influences shown in this figure are hard to figure out directly. Some of them are combined with each other, which make it too complicated to explain the relationship between the sources of errors and the output voltages.

The following calibration steps were carried out on a computer with the help of Matlab software. By substituting the measured data sets to Equation (10), the unknown parameters can be figured out. Section 3 introduces the calibration results and our analysis.

## 3. Results and Discussion

Using the parameter substitution and curve fitting method introduced in Section 2.2, the eight unknown parameters were calculated, which are listed in Table 1. The offset voltages $B_{1},B_{2}$ and amplifying factors $A_{1},A_{2}$ are close to the theoretical values, while the orientations of the four polarizers ($\phi_{1},\phi_{2},\phi_{3},\phi_{4}$) show significant differences compared with the designed values. Because the polarizers were mounted manually, the orientation error might become out of control, which emphasized the importance of the calibration of the mounting errors. In fact, the manufacturing processes might be able to achieve a greater accuracy. The calibration method was tested under different mounting errors. The calibration method performed as well as the case presented in this paper. In this way, our method was proved to be robust to the size of mounting errors.

**Figure 6:**
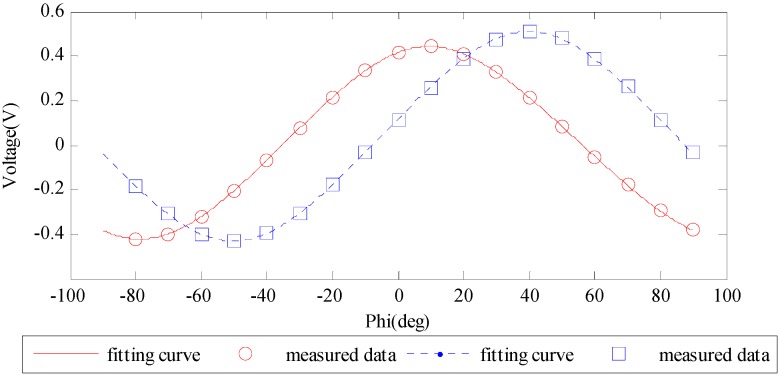
The fitting curve of the output voltage of the two groups of orthogonal channels. The red line and circles represent the fitting curve and measured output voltage of channels 1/3, the blue dashed line and squares represent channels 2/4.

One period of the fitting curve of the output voltage is shown in Figure 6 together with the measured voltage data.

**Figure 7:**
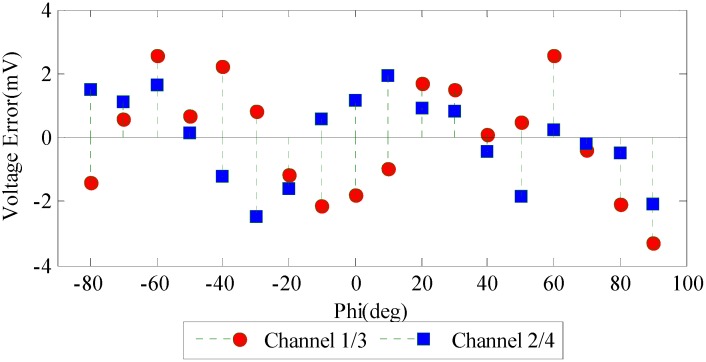
The error between the fitting curve and output voltages. The remaining voltage errors are within ±4 mV which are due to the output offset voltages of the two log ratio amplifiers (typical value ±3 mV) and the noises of other circuit components. This random voltage error is hard to compensate.

From Figure 6 we can find that the amplitudes of the two curves are different from each other, which is mainly due to the differences of the electric components of the two groups of orthogonal channels such as the gain factors, offset voltages and amplification factors. The normalization method has frequently been used by similar systems in processing the output voltage data [20,21,23,31]. However, some error sources might be ignored and the calibration process might become complicated. Using the curve fitting method mentioned in this paper, the normalization is no longer a necessary process and the calibration becomes simplified. The errors between the fitting voltage curve and the measurements are shown in Figure 7.

Figure 6 and Figure 7 show that the output voltage curves can be accurately fitted using the method proposed above. After substituting the eight unknown parameters with the estimated values in Table 1, Equation (10) can well describe the relationship between incident light AOP and output signal. The voltage error is limited within ±4 mV which means the new mathematical model is a good fit to the sensor output. 

The unknown parameters in Equation (10) become fixed values after calibration. When a pair of output voltage was obtained, the AOP and DOP of incident light can be figured out using Equations (5) and (11). By comparing the measured polarization information (AOP and DOP) with the real state of incident light, the measurement error can be evaluated.

**Figure 8:**
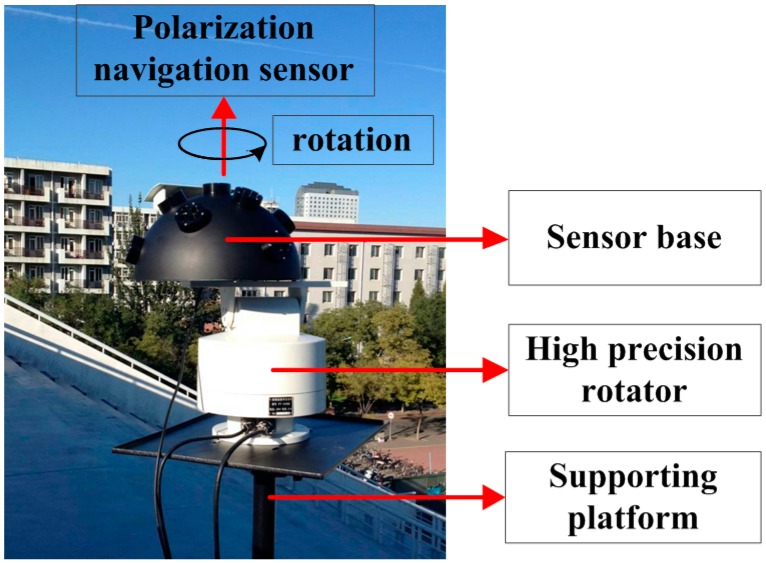
The experiment under clear sky. The tested sensor was aimed at the zenith of the sky dome. It was carried by a high precision rotator which was controlled by a computer. The sensor base was used to fix the orientation of the sensor.

The sensor was tested under clear sky conditions, because the DOP of scattered skylight was relatively high and the polarization state is relatively stable and predictable. The sensor was aimed at the zenith of the sky dome and carried by a high precision numerical controlled rotator. The rotator rotated around the vertical axis and the angular resolution is 0.0125°. It was controlled by a computer through the serial port communication. During the test, the sensor was rotated 180° with a step of 10° (from −90° to 90°). At each step, the AOP and DOP data measured by our sensor were collected by a laptop through the universal serial bus communication. A whole group of tests took about 10 min. Although the DOP and AOP of the skylight changed a little during the test, compensation has been made to correct the solar movements. The experiment was carried out on 12 October 2015 in Beijing, China (geographic coordinates: 116.358415° E, 39.987009° N). The data in this paper was collected from 8:19:22 to 8:28:43 (UTC, hour: minute: second). During the measurement, the solar azimuth angle and solar zenith angle changed by about 1.658°. The results measured by the sensor were compared with the rotator to validate the AOP error. Limited by the instrument, we cannot get a reliable measurement of the accurate DOP of the skylight. The theoretical model based on Rayleigh scattering cannot offer us the accurate DOP of the skylight either. So the measurement of DOP was evaluated by the stability at a different rotation angle. Figure 8 shows the experiment scene.

**Figure 9:**
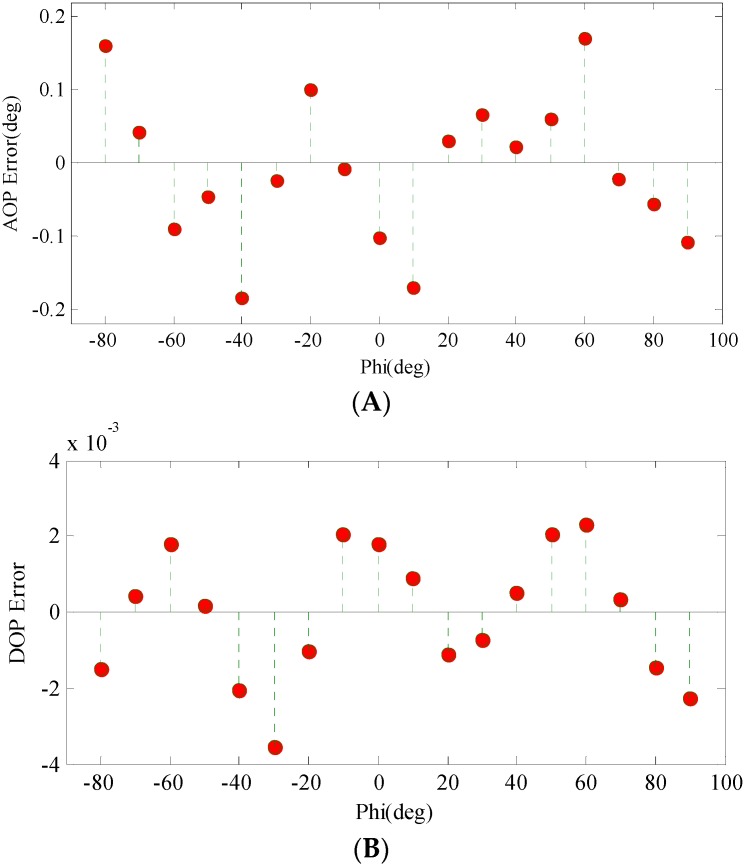
The measurement errors of AOP (**A**) and DOP (**B**). The AOP errors are relatively low and the DOP measurements are stable. The potential remaining sources of error may come from the random output noises of the circuit components, the analog to digital converter (ADC) errors and the unstable incident light.

The measurement errors of AOP and DOP are shown in Figure 9A,B, respectively. Figure 9A shows that the AOP error of the polarization navigation sensor is within ±0.2°. The accuracy reaches a high level; moreover, it needs no compensation. The AOP errors are lower than many previous studies. For example, the output error of AOP was about ±1.5° in Lambrinos’ work [20], the polarization navigation sensor developed by Zhang reached about ±0.3° [27]. The six channel sensor developed by Chu [21] obtained an accuracy of ±0.2° which is the same as ours. The potential remaining sources of error may come from the random output noises of the circuit components. The analog to digital converter could also introduce errors. In addition, the incident light was not absolutely stable and reliable, which might introduce errors to the measurement results. Figure 9B shows that the stability of DOP can be limited within ±0.4%. However, the accuracy of DOP cannot be determined yet. The evaluation of the accuracy of DOP needs other independent measurements with a reliable instrument. During the calibration experiment, the DOP of the outgoing light was fixed near 1. Theoretically, the variance of DOP will influence the amplitude of the output voltage. For example, the amplitude of the voltage in Figure 5 will decrease when the DOP decreases. However, the DOP will not change the phase of the output voltage curve. In Chu’s study, the output voltages under different DOPs were analyzed: “The signals become independent of the degree of polarization after the normalization process and are practically identical” [21]. In fact, the decrease of DOP will cause an accuracy loss in the AOP measurement. In our outdoor experiment, the DOP of the zenith was about 0.58. The measured AOP error was ±0.2°, which means that the calibration results are still reliable under this situation.

During the polarization navigation, if the AOP of the sky zenith was acquired with our sensor, the solar azimuth angle could be derived with an error of ±0.2°, theoretically. The direction information can be further derived by considering the geographical location and skylight polarization pattern. However, the polarization patterns of skylight could be seriously changed by the atmosphere condition, such as the aerosol, cloud, smoke, etc. For now, the sensor can be appropriately applied to the clear sky conditions.

## 4. Conclusions

Aiming at the application of polarization navigation, this paper proposed a point source polarization sensor with four parallel channels. The working principle of the sensor was introduced from structure design and signal processing. It was a classic bio-inspired polarization sensor with several improvements in the design and calibration method. Most of the error factors had been considered in the construction of the mathematical model. Since it is hard to directly measure these error elements, we proposed a calibration method using parameter substitution and curve fitting. The mathematical model of the sensor was greatly simplified after a substitution transformation. The calibration experiment has been carried out. A curve fitting method was used to figure out the unknown elements according to the sensor’s mathematical model. The results showed that the fitted curve is consistent with the measured data. After calibration, the measurement error of AOP was limited within ±0.2° without compensation. The measurement stability of DOP can be controlled within ±0.4%. Besides, the compensation for solar movement is necessary during an outdoor experiment. In our future work, we will try to expand the single detector into a multi-directional sensor, which could collect the polarization information from several directions of the sky hemisphere synchronously, so that the navigation function of an insect’s compound eye could be better realized.

## Figures and Tables

**Table 1:** The calibration results of the unknown parameters.

$B_{1}$	$A_{1}$	$\phi_{1}$	$\phi_{3}$
0.01064	0.97869	12.656	98.541
$B_{2}$	$A_{2}$	$\phi_{2}$	$\phi_{4}$
0.03950	1.06194	39.919	130.010

## Abbreviations

The following abbreviations are used in this manuscript:

CCD	charge coupled device
CMOS	complementary metal oxide semiconductor
AOP	angle of polarization
DOP	degree of polarization
DRA	dorsal rim area
IFOV	instantaneous field of view
FPGA	field programmable gate array
FOV	field of view
SNR	signal noise ratio
ASD	analytical spectral device
UTC	Coordinated Universal Time
POL-neuron	polarization-opponent neuron
ADC	analog to digital converter
